# Simulator-based ultrasound training for identification of endotracheal tube placement in a neonatal intensive care unit using point of care ultrasound

**DOI:** 10.1186/s12909-020-02338-4

**Published:** 2020-11-07

**Authors:** Khushboo Qaim Ali, Sajid Bashir Soofi, Ali Shabbir Hussain, Uzair Ansari, Shaun Morris, Mark Oliver Tessaro, Shabina Ariff, Hasan Merali

**Affiliations:** 1 Department of Pediatrics & Child Health, Aga Khan University, Karachi, Pakistan; 2 Division of Infectious Diseases, Department of Pediatrics, The Hospital for Sick Children, Toronto, Canada; 3 Pediatric Emergency Medicine, Emergency Point-of-Care Ultrasound Program, The Hospital for Sick Children, Toronto, Canada; 4 Pediatric Emergency Medicine, McMaster Children’s Hospital, Hamilton, Canada

**Keywords:** Simulation, Ultrasound training, Endotracheal tube, Point of care ultrasound, Resuscitation, Neonates

## Abstract

**Background:**

Simulators are an extensively utilized teaching tool in clinical settings. Simulation enables learners to practice and improve their skills in a safe and controlled environment before using these skills on patients. We evaluated the effect of a training session utilizing a novel intubation ultrasound simulator on the accuracy of provider detection of tracheal versus esophageal neonatal endotracheal tube (ETT) placement using point-of-care ultrasound (POCUS). We also investigated whether the time to POCUS image interpretation decreased with repeated simulator attempts.

**Methods:**

Sixty neonatal health care providers participated in a three-hour simulator-based training session in the neonatal intensive care unit (NICU) of Aga Khan University Hospital (AKUH), Karachi, Pakistan. Participants included neonatologists, neonatal fellows, pediatric residents and senior nursing staff. The training utilized a novel low-cost simulator made with gelatin, water and psyllium fiber. Training consisted of a didactic session, practice with the simulator, and practice with intubated NICU patients. At the end of training, participants underwent an objective structured assessment of technical skills (OSATS) and ten rounds of simulator-based testing of their ability to use POCUS to differentiate between simulated tracheal and esophageal intubations.

**Results:**

The majority of the participants in the training had an average of 7.0 years (SD 4.9) of clinical experience. After controlling for gender, profession, years of practice and POCUS knowledge, linear mixed model and mixed effects logistic regression demonstrated marginal improvement in POCUS interpretation over repeated simulator testing. The mean time-to-interpretation decreased from 24.7 (SD 20.3) seconds for test 1 to 10.1 (SD 4.5) seconds for Test 10, *p* < 0.001. There was an average reduction of 1.3 s (β = − 1.3; 95% CI: − 1.66 to − 1.0) in time-to-interpretation with repeated simulator testing after adjusting for the covariates listed above.

**Conclusion:**

We found a three-hour simulator-based training session had a significant impact on technical skills and performance of neonatal health care providers in identification of ETT position using POCUS. Further research is needed to examine whether these skills are transferable to intubated newborns in various health settings.

**Trial registration:**

ClinicalTrials.gov Identifier: NCT03533218. Registered May 2018.

## Background

Simulator-based medical education (SBME) is emerging as a popular method for improving health care provider knowledge and performance in a variety of settings [1, 2]. Simulation-based training has been shown to decrease medical errors and provide a platform to enhance learner knowledge, clinical skills and performance in a controlled environment that translates to improved patient care [3–8]. SBME is a learner-centered approach that enhance clinical technical skills through repeated practice and reflection before performing these techniques on patients [9–11]. A variety of simulators are presently used in areas of medical training involving invasive procedures such as lumbar puncture, central line insertion, abscess drainage, airway management and cardiopulmonary resuscitation [9, 12].

Numerous homemade simulators have been used for training in interventional skills such as central venous access, chest tube insertion,umbilical artery catheterization and airway procedures such as tracheostomy and cricothyrotomy [9]. These simulators have been assembled using easily acquired local materials such as closed-cell extruded polystyrene foam (Styrofoam), Ziploc bags and plastic bottles [9]. Low-cost “homemade” simulators are inexpensive and easily reproducible [9]. The 2030 Lancet Commission on Global Surgery meeting also suggested the use of locally available and affordable models for training in low- and middle-income countries (LMICs) [13]. These cost-effective models have proven efficacious for simulation-based training in achieving expertise in many clinical tasks [11] including Point of care ultrasound (POCUS) [14].

POCUS is a powerful diagnostic modality in both adult and pediatric critical care settings and is an accurate and rapid method of detecting tracheal or esophageal intubation, with an average performance time of 9 s [15, 16]. The technique is easy to learn and can be performed by novice operators [15]. This user-friendly method has a high sensitivity (93–100%) and specificity (97–100%) in adults and children [17, 18]. POCUS has substantial advantages over standard methods for confirmation of endotracheal tube position, such as the ability to be performed without interruption during resuscitation [19].

**Fig. 1:**
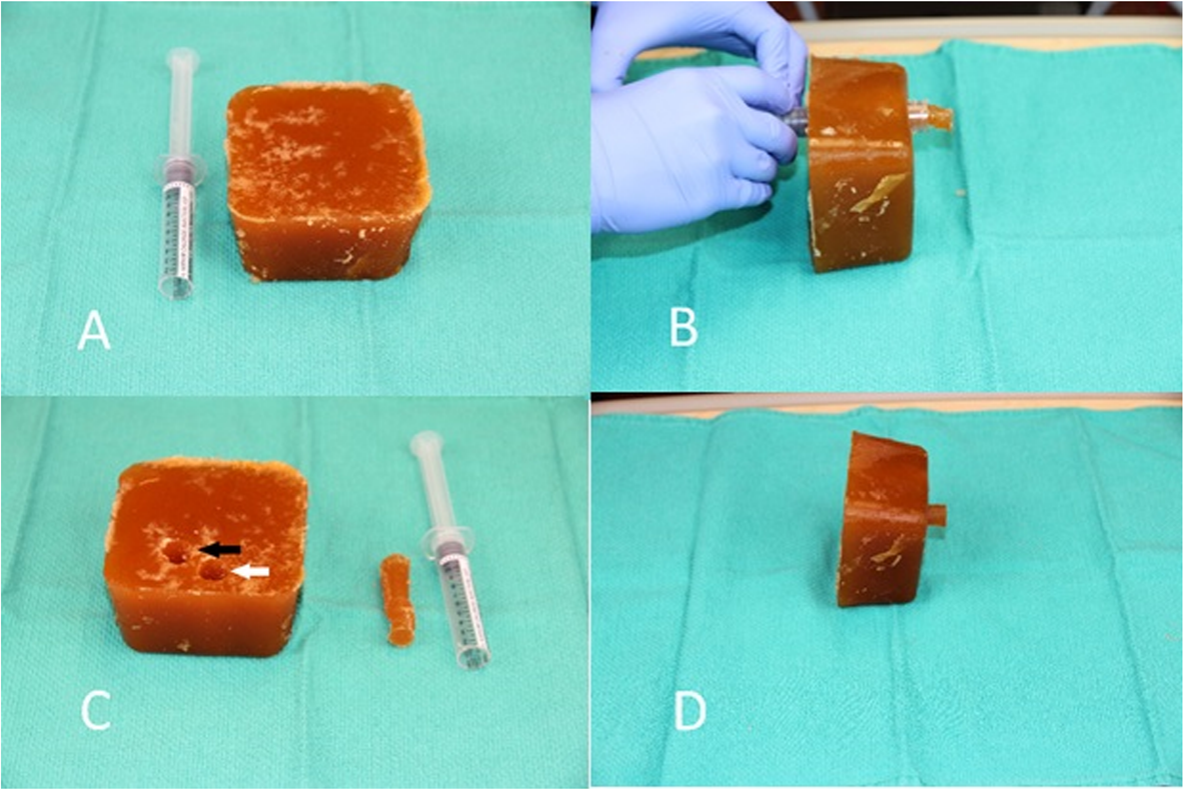
Low cost ultrasound simulator. **a** The beef gelatin and psyllium fiber block, with cut-off 10 mL syringe. **b** Syringe barrel being used to create 2 lm within the block. The plug created within the syringe by passage of the syringe through the block is then expelled from the syringe by depressing the plunger – one such plug is displayed next to the now empty syringe. **c** The white arrow indicates the simulated trachea, while the black arrow indicates the simulated esophagus. **d** The completed simulator, with a plug partially inserted into the simulated esophagus. When the plug is fully inserted, the simulator produces the sonographic appearance of a tracheal intubation. When the plug is removed, the simulator produces the sonographic appearance of an esophageal intubation

We have previously described in detail the novel simulator that we used during the training sessions. Details of the simulator is presented in the previously published POCUS protocol paper [20]. The simulator can mimic the sonographic appearance of the anterior neck for both a correctly placed (tracheal) endotracheal tube (ETT) and an incorrectly placed (esophageal) ETT (Fig. 1).

In this study, we investigated the usefulness of a low-cost, novel POCUS simulator to train health care providers with minimal or no POCUS experience to accurately detect tracheal versus esophageal intubation in neonates. We also investigated whether the time to POCUS image interpretation decreased with repeated simulator attempts.

## Methods

The study was carried out in the Neonatal Intensive Care Unit (NICU) of the Department of Pediatrics & Child Health at the Aga khan university Karachi between May and June 2018.

We invited health care providers who worked in the NICU and were interested in participating in the study. An informed consent was obtained from all participants. Sixty health care providers consented to participate. They included neonatal attending physicians (17%), neonatal and pediatric intensive care fellows (15%), pediatric residents (45%) and senior neonatal nurses (23%) respectively. Demographic data of the participants was collected including age, gender, professional designation, and years of clinical experience, average numbers of deliveries attended and average intubations performed /month. We also collected information on any previous knowledge and experience in POCUS for airway and other critical procedures.

Six sessions were conducted, each 3 h in duration. The training sessions were facilitated by study investigators with pediatric intubation POCUS expertise. All POCUS was performed using a Lumify 12-2 MHz linear ultrasound transducer (PHILIPS Ultrasound, Inc., Bothell, Wash., USA) connected to an android version, Galaxy Tab. A (2016) SM-T285, with a depth setting of 2.5 cm and a preset imaging of 3 s.

The study was approved by the Research Ethics Boards at The Hospital for Sick Children (REB #1000057021), the AKUH Ethical Review Committee (ERC#: 4927-Ped-ERC-17) and the National Bioethics Committee of Pakistan (4–87/NBC-319/18/552).

### Simulator-based training

**Fig. 2:**
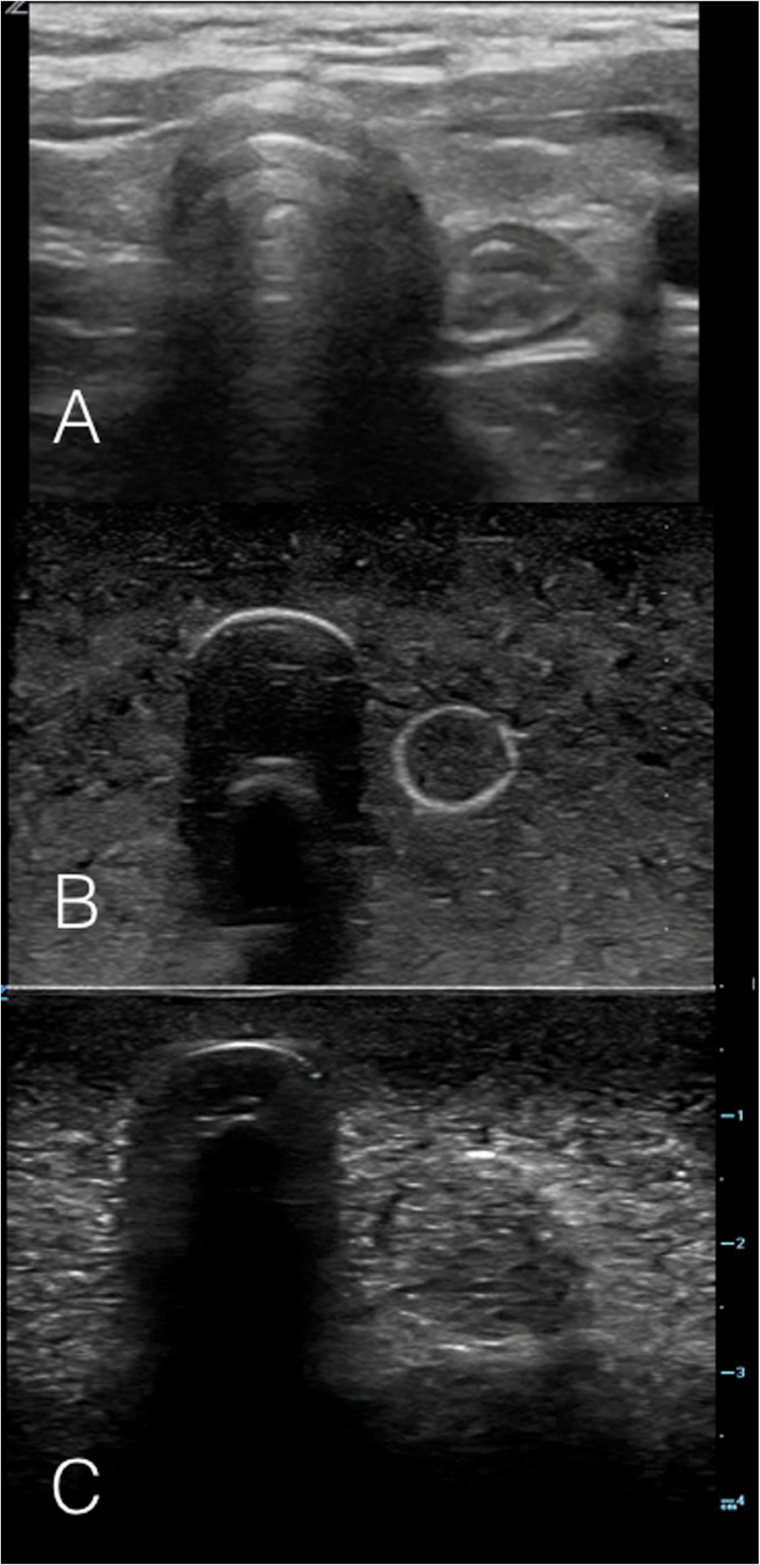
Ultrasound images on the Simulator model Tracheal Intubation. **a** Static ultrasound image of a patient with the ETT in the trachea, with linear ultrasound probe held in transverse orientation over the anterior neck at the level of the sternal notch. **b** & **c** Static ultrasound images of beef gelatine model with plug inserted into simulated esophagus, simulating the ultrasound appearance of a tracheal intubation

**Fig. 3:**
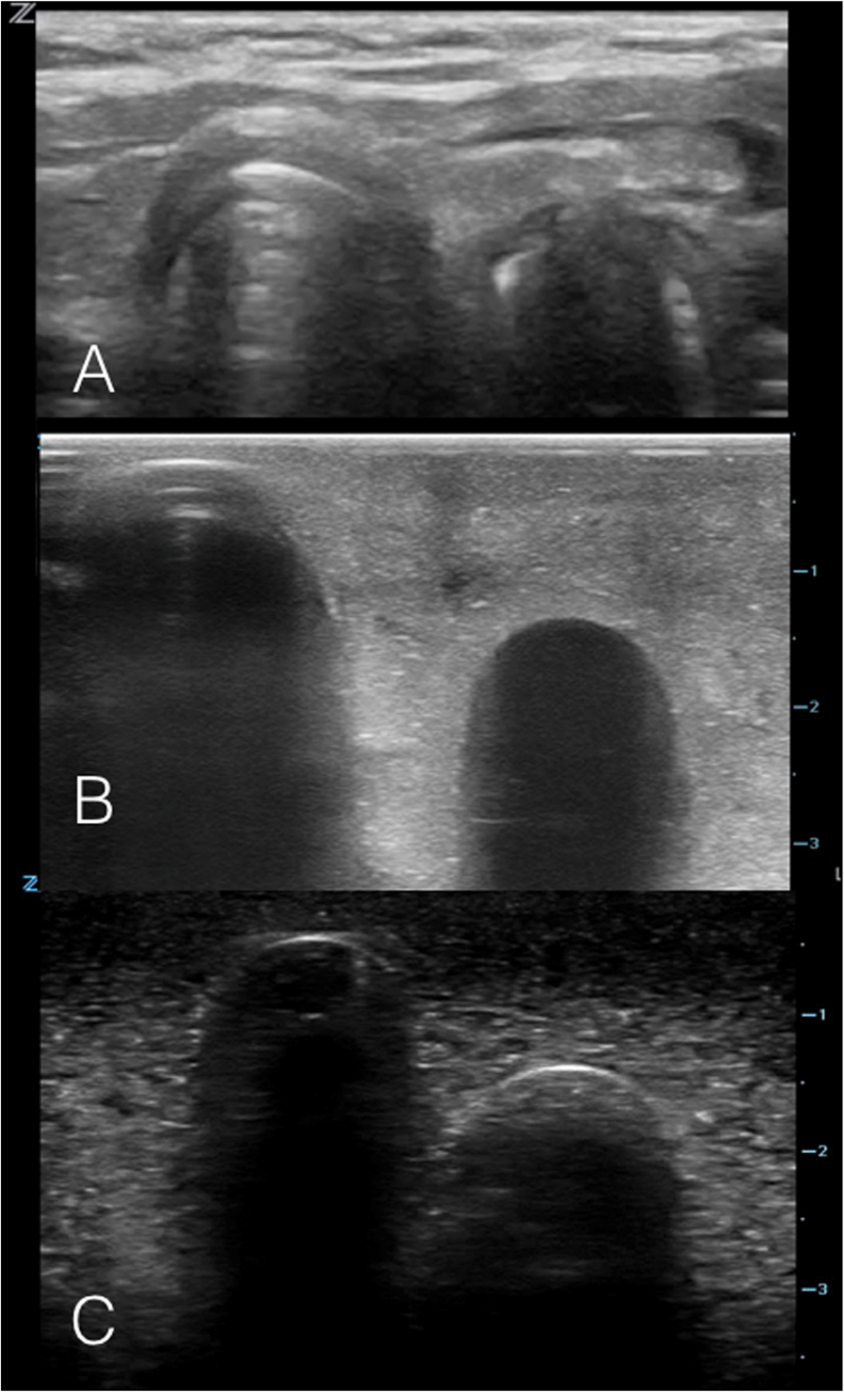
Ultrasound images on the model: Esophageal Intubation. **a** Static ultrasound image of an esophageal intubation in a patient, with linear ultrasound probe held in transverse orientation over the anterior neck at the level of the sternal notch. **b** & **c** Static ultrasound images of beef gelatin model with plug removed from simulated esophagus, simulating the ultrasound appearance of an esophageal intubation

The simulator-based training sessions were designed to provide participants with conceptual knowledge and capacity building prior to performing POCUS on stable intubated NICU patients. Each session consisted of three components: 1) a didactic session, 2) hands-on practice (on the simulator) 3) a demonstration on stable NICU patients and finally 4) skill evaluation. The didactic session included a presentation, question and answer session, and an interactive simulator demonstration. During the presentation, participants were taught to recognize relevant anterior neck sonographic anatomy (trachea, esophagus, carotid arteries and thyroid gland), as well as the POCUS sonographic appearance of tracheal and esophageal intubations. (Figs. 2 and 3).

The simulator demonstration began by using a neonate-sized doll to show appropriate transducer orientation and placement at the anterior neck for intubation POCUS. A stepwise POCUS method was taught, including entering subject information, application of appropriate amount of gel, correct transducer positioning, turning on the POCUS application on the tablet, capturing images and probe cleaning.

The simulator demonstration focused on recognition of the sonographic appearances of tracheal and esophageal intubation. Two simulator models were used, one simulating a tracheal intubation (i.e. plug inserted in the simulated esophagus) and the other simulating an esophageal intubation (i.e. plug removed from the simulated esophagus) [21].

At the completion of the demonstration, each participant was given an hour for hands-on practice with the simulators to recognize tracheal and esophageal intubations using POCUS. Participants then demonstrated their skills on stable intubated NICU patients (whose parents provided informed consent prior to procedure). POCUS was performed by the participants on these neonates to evaluate the participant’s ability to translate their skills to real patients. It was not feasible logistically, nor was it ethical to perform multiple POCUS attempts on live patients due to the nature of the NICU population. However, each participant was examined and observed at least once for recognition of major landmarks and for compliance of each step taught in POCUS protocol. The master trainers provided individual and group feedback to the trainees on their bed side skills of POCUS. However, we did not document or rate the performance of individual participants on real patients.

Following training the participants underwent an objective assessment facilitated by POCUS experts. The experts were university faculty members with rich experience in various medical evaluations including OSAT. A 13-item objective structured assessment of technical skills (OSATS) on two different simulators via POCUS was carried out. (See Appendix 1) OSATS have previously been validated as a method of assessing POCUS skills [22, 23].

We sought a competency level of at least 75% and above in the OSAT as a prerequisite to proceed to the second phase of POCUS training. Participants receiving a score of 10 or higher (out of 13) in the OSAT were then evaluated on their ability to differentiate simulated tracheal and esophageal intubations. The participants underwent 10 tests on the hand -made Simulator. We choose to do 10 attempts as this was the maximum allowable attempts given the time constraints of the training session.

For each simulator test, a random number generator from Google (Google LLC, CA, USA, 2017) was used to decide whether the simulator would be placed in the tracheal or esophageal intubation position with participants blinding ensured with surgical drapes placed over the simulator [21]. For each of the tests, a timer was used to record the time elapsed until participant interpreted the POCUS images.

At the end of training we collected feedback from the participants through a structured questionnaire. The questionnaire comprised of two components; one on the assessment of tools and methods used for training and the second on the overall effectiveness of the training exercise with the simulator (See Appendix 2).

## Statistical analysis

Data analysis was performed using Stata (V.15, Stata Corp, TX, USA, 2017). Descriptive statistics were reported as mean (SD) for quantitative variables and frequency and percentage for categorical variables. The primary outcomes were: 1) percentage of participants interpreting the simulator correctly for each simulator test and 2) time-to-interpretation for each simulator test.

The correct decision referred to correct responses on all of three domains evaluated including the number of air columns seen, esophageal appearance (empty or full) and the final interpretation of ETT position. We used the paired t-test to compare the mean time taken for correct interpretation of the simulated images between the first and the tenth test.

We used mixed-effects logistic regression to determine factors that had a significant impact on correct interpretation over repeated rounds of simulator-based training. Repeated measures analysis was performed through a linear mixed model to evaluate factors that were associated with time-to-interpretation. We adjusted the models for gender, profession, years of experience, and baseline POCUS training. Results were reported as odds ratios (OR) for the mixed-effects logistic regression model and beta coefficients for the linear mixed model.

## Results

Participant demographic data are shown in Table 1. The majority of participants were pediatric residents (45%) and the average years of neonatal care experience of all subjects was 7.0 (SD = 4.9). Only 6 (10%) participants had received prior POCUS training.

**Table 1:** Characteristics of Participants

	Total *N* = 60
**Gender**	
Female	35 (58%)
Male	25 (42%)
**Age Categories**	
25–30	14 (23%)
31–34	9 (15%)
> 34	10 (17%)
Missing	27 (45%)
Age, mean ± SD	32.7 (4.8)
**Years of experience (**mean ± SD)	7.0 (4.9)
Average # of neonates (28 days and younger) cared each month	
< 10	12 (20%)
11–50	22 (37%)
50+	25 (42%)
None	1 (2%)
Average deliveries attended per month	
< 5	13 (22%)
5–20	27 (45%)
20+	15 (25%)
Not Applicable	5 (8%)
Average neonatal intubations performed per month?	
< 10	32 (53%)
11–50	23 (38%)
50+	3 (5%)
None	2 (3%)
Awareness regarding POCUS prior to training	
Yes	22 (37%)
No	38 (63%)
Received training on point of care ultrasound	
Yes	6 (10%)
No	54 (90%)
Received training on airway ultrasonography	
Yes	2 (3%)
No	58 (97%)
	Total N = 60

Majority of participants 59/60 scored ten and above in the OSAT. Assessment of technical skills illustrated that majority 59 (98%) participants had sound understanding of POCUS skills. Skills that participants scored highest on were hand washing (100%), organization of equipment (100%), turning on POCUS device (100%) and entering a subject identifier in the POCUS device (100%). The next highest scored skills were placement of POCUS device relative to manikin (97%), appropriate transducer orientation (97%), transducer cleaning after scanning (97%), appropriate volume and distribution of gel on the simulator (98%) and appropriate POCUS scan coverage of manikin neck (97%). The least learned technical skills observed were appropriate gain settings and image recording, which scored 83 and 87%, respectively. (Table 2).

**Table 2:** Results of objective structured assessment of technical skills (OSAT)

	Correctly done count (n = 60)	%
1 Appropriate gain settings	48	80%
2 Images appropriately recorded	51	85%
3 Transducer cleaning prior to scanning	55	92%
4 Appropriate depth settings	55	92%
5 Turning off the POCUS device	57	95%
6 Placement of the POCUS device relative to the manikin to allow easy visualization of both	58	97%
7 Appropriate transducer orientation	58	97%
8 Transducer cleaning after scanning	58	97%
9 Demonstrated POCUS scan covers appropriate areas of manikin’s neck	58	97%
10 Appropriate volume and distribution of gel on simulator	59	98%
11 Preparation for procedure Hand Washing / Gloves / organize equipment	60	100%
12 Turning on the POCUS device	60	100%
13 Entering subject (manikins) identifier in POCUS device	60	100%

**Fig. 4:**
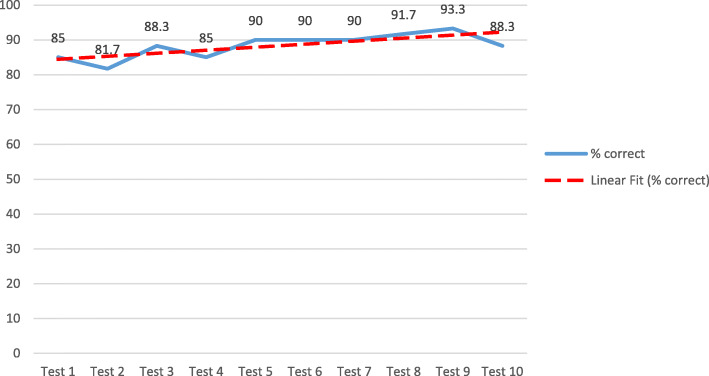
Aggregate correct identification and interpretation of simulator ETT position with each test

On the competency assessment of POCUS performance test, 51 (85%) participants correctly interpreted the images on Test 1 and, by Test 5, scores improved to 90%. We observed retention of correct interpretation from Test 5 to Test 9. A slight reduction in score was observed after Test 9, suggesting that Test 9 was the saturation point (Fig. 4).

After controlling for the effects of gender, professional designation, years of clinical experience and baseline POCUS training, the multivariate analysis showed marginal improvement in correct interpretation of the simulated images with repeated testing. However, this finding was not statistically significant (OR = 1.09; 95% CI: 0.998–1.19) (Table 3).

**Table 3:** Multivariate analysis to identify the impact of intervention on correct decision and interpretation time

	Multivariate (Outcome Time)				Multivariate (Outcome Correct Decision)			
Parameter	Coef.	95% CI		P	OR	95% CI		P
**Test**	−1.33	−1.66	−1.00	< 0.0001	1.09	0.998	1.19	0.056
**Gender**								
Female	Ref.				Ref.			
Male	−0.57	−2.78	1.63	0.609	0.60	0.33	1.06	0.079
**Professional Designation**								
Neonatal fellows/ Neonatal attending physicians	−1.70	−4.42	1.01	0.219	1.07	0.53	2.15	0.845
Postgraduate medical trainees	1.07	−1.78	3.91	0.462	1.68	0.81	3.48	0.165
Senior nursing staff	Ref.				Ref.			
**Years of experience in newborn care**	0.18	−0.06	0.43	0.140	1.07	0.999	1.15	0.052
**Trained in airway ultrasonography**								
Yes	Ref.				Ref.			
No	−3.46	−8.48	1.56	0.177	(omit)*			

* Outcome in the data is same for trained individuals thus, omitted from model

**Fig. 5:**
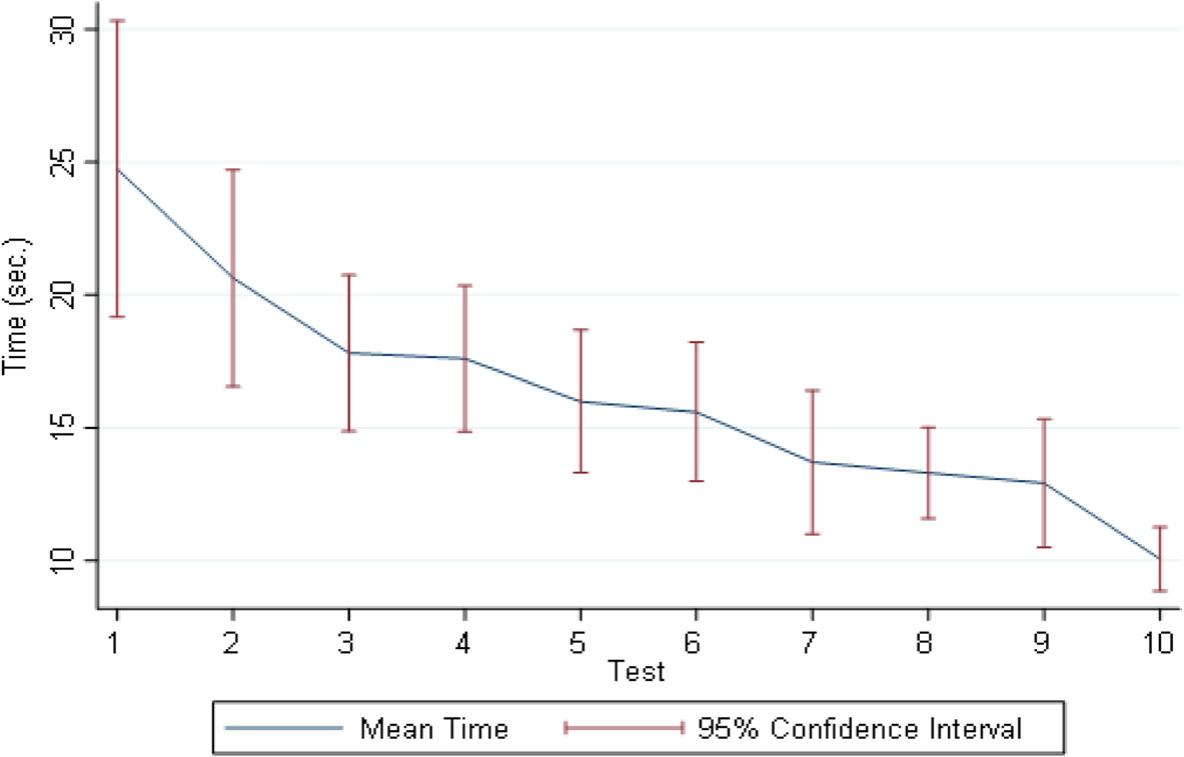
Time-to-interpretation (in seconds) with 95% CIs

The mean time-to-interpretation trended lower with repeated testing, from a high of 24.7 (SD 20.3) seconds for Test 1 to a low of 10.1 (SD 4.5) seconds for Test 10, *p* < 0.001 (Fig. 5). After adjusting for the covariates listed above, the time-to-interpretation was found to decrease by an average 1.3 s (β = − 1.3; 95% CI: − 1.66 to − 1.0) for each round of simulator-based testing.

We also collected feedback from the participants on their experience of training and POCUS procedure. Written comments suggested positive feedback from the participants. 95% of participants rated the training as “very easy” and 97% of participants reported that the visual aids were “very effective”. More than half of the participants (54%) were very satisfied with the hands-on training session. The participants shared that sonographic explanation via the simulator, live demonstration on NICU babies and hands-on training were the best parts of the training. Participants also expressed that the training was an informative initiative since it had practical implications. Some participants faced difficulty in identifying air columns and in the interpretation of POCUS images. They also found it difficult to hold the Lumify transducer correctly and position it with adequate depth. However, the overall feedback was positive and the training was considered comprehensive, well organized and an excellent learning experience.

## Discussion

Our study showed a 3-h session with a novel low-cost simulator was an effective method of training a diverse cadre of health care providers in POCUS. The exercise of repeated interpretation with the aid of simulator led to a significant improvement in time to interpretation of 1.3 s for subsequent testing. Overall, from test 1 there was a high of 24.7 s to interpretation which decreased to 10.1 s by test 10. Decreasing interpretation time in the context of neonatal intubations can have a meaningful impact on clinical outcomes in newborns and lead to quick decisions and prompt management, critical in the labor room and emergency settings. The immediate postnatal period is the most vulnerable time in a newborn’ life where the majority of intubations take place to assist in the transition from fetal to neonatal life. Prompt interventions can prevent hypoxia and irreversible damage to the newborn’s developing brain [20].

In recent years, several studies have documented the effectiveness of simulation-based learning, especially for procedural skills [24]. One benefit of simulation-based training highlighted is better comprehension, which may in part be due to decreased levels of anxiety in learners [25]. Others studies have shown low-cost POCUS simulators improve skills for POCUS novices [26–28]. Parks, in 2013, carried out standardized ultrasound competency training through simulation that included online teaching, didactic presentations and hands-on practice. This study demonstrated improvement in learners’ competency level to correctly interpret POCUS images [14]. Similarly in 2018, Jensen et al., evaluated 25 novice ultrasound trainees in simulator based training for Focused Assessment with Sonography for Trauma (FAST) exams, followed by a practice phase that resulted in 80% trainees achieving mastery level [29]. Abelson showed a correlation between repeated simulations and a stronger effect on acquiring high-level clinical skill [24].

Apart from developing healthcare professionals’ knowledge and skills, stimulation has also provided an opportunity to improve patient outcomes and protect them from unnecessary risks. Khouli et al. (2011) trained second and third year residents on central line insertion using traditional video method and simulator plus video method. The incidence of catheter-related infections reduced to 70% in the simulator group [30]. In the same vein, results of a meta-analysis by McGaghie et al. (2011), showed that stimulator based training is associated with better outcomes in gynecological surgeries and neonatal outcomes (in patients with shoulder dystocia, and a reduction of neonatal injury) [31].

There are several features of simulation that lead to effective learning which we incorporated into this training including clearly defining outcomes, repetitive practice with simulator, providing individual feedback on skills and simulation clinical variation, learning in a controlled environment, and individualized learning with the simulator [32]. Additional best practices described in the literature include integrating this simulation into the standard medical/nursing curriculum, practicing with increasing levels of difficulty, adapting the simulator to complement multiple learning strategies and ensuring the simulator is a valid learning tool [32].

Simulation based trainings are relatively inexpensive and provide an environment for skills to be obtained quickly. However the long term impact on the skills of the trainees are dependent upon refresher trainings and assessments. Smith in 2010 assessed the long term impact of simulated sessions and showed a significant decline of skills over time without refresher training and assessments. He compared the trainees with the novice group after 3 months of simulated training session and found no difference in competency in both groups. Hence a single simulated session may serve to improve procedural skills but it is insufficient to sustain the competency and ongoing refreshers, frequent monitoring and feedback are critical [33]. Similarly Shah et al. in 2017, evaluated the effectiveness of a medium fidelity simulator for training seventy-six third year medical students in normal vaginal delivery. The students were divided into two groups; group A was taught with the traditional method (PowerPoint presentations), while group B was taught with simulation. Post training assessment showed better skills retention in group B compared to their counterpart [34].

This study has several limitations. The training was conducted at a single institution over a short time period, limiting the generalizability of our results. Participants reported a range of baseline POCUS knowledge and we did not perform a pre-test for POCUS skill assessment. This heterogeneity may have impacted results and learning outcomes. This study also did not address the effects of this simulation-based intervention on patient care and satisfaction. One cannot rule out social desirability bias in the feedback. We attempted to reduce the bias by keeping the identity of the feedback trainees anonymous. The data was handled by research coordinator who were not affiliated with clinical care in the NICU.

Despite these limitations, our study demonstrates that a novel, low-cost simulator is an effective tool to use in skill enhancement for interpretation of sonographic images, and for teaching the correct interpretation of ETT placement to newborn providers. Importantly, development of airway POCUS skills was made possible with the simulator, without which training would neither be feasible nor ethical.

## Conclusions

A 3-h simulator-based training session appears to be effective in teaching intubation POCUS to a variety of neonatal providers. We found improvements in accuracy and time-to-interpretation with repeated simulator use. Further studies are required to investigate the potential clinical benefit of simulator-based POCUS training in neonatal settings.

## Recommendations

POCUS training using low cost simulator should be incorporated in all neonatal training programs to achieve intubation POCUS competency. Low-cost POCUS simulators have the potential to allow such trainings to be conducted in low-resource settings. This will improve the quality of care and has the potential to reduce exposure to radiation with repeated X-rays. These low-cost simulator trainings need to be scaled up to level 2 care facilities to minimize financial burden, radiation exposure and specialized manpower (radiologist). Future studies are required to explore POCUS for evaluating the depth of ETT.

## Data Availability

The data available is available on request.

## Appendix 1

**Table 4:** Objective Structured Assessment of Technical Skills

Preparation for procedure Hand Washing/Gloves/organize equipment	0............ Not done OR done incorrectly 1............ Done correctly
Transducer cleaning prior to scanning	0............ Not done OR done incorrectly 1............ Done correctly
Turning on the POCUS device	0............ Not done OR done incorrectly 1............ Done correctly
Entering subject (mannikin) identifier in POCUS device	0............ Not done OR done incorrectly 1............ Done correctly
Placement of the POCUS device relative to the mannikin to allow easy visualization of both	0............ Not done OR done incorrectly 1............ Done correctly
Demonstrated POCUS scan covers appropriate areas of mannikin’sneck	0............ Not done OR done incorrectly 1............ Done correctly
Appropriate volume and distribution of gel on simulator	0............ Not done OR done incorrectly 1............ Done correctly
Appropriate gain settings	0............ Not done OR done incorrectly 1............ Done correctly
Appropriate depth settings	0............ Not done OR done incorrectly 1............ Done correctly
Appropriate transducer orientation	0............ Not done OR done incorrectly 1............ Done correctly
Images appropriately recorded	0............ Not done OR done incorrectly 1............ Done correctly
Turning off the POCUS device	0............ Not done OR done incorrectly 1............ Done correctly
Transducer cleaning after scanning	0............ Not done OR done incorrectly 1............ Done correctly
Overall performance	1…………………..Competent 2…………………..Not competent

## Appendix 2

**Table 5:** Learner Feedback Form

Regarding your training on Point-of-care ultrasound:	
1 How easy was it to understand?	1 Very easy
	2 Mostly easy
	3 Easy
	4 Somewhat easy
	5 Not easy
2 How effective were the visual aids used?	1 Very effective
	2 Mostly effective
	3 Somewhat effective
	4 Slightly effective
	5 Not effective
3 Were you satisfied with the hands-on training session?	1 Extremely satisfied
	2 Very satisfied
	3 Moderate satisfied
	4 Slightly
	5 Not at all satisfied
Regarding your application of the **Point-of-care ultrasound on real patients**	
4 Did your training adequately prepare you?	1 Extremely adequately
	2 Very adequately
	3 Adequately
	4 Somewhat adequately
	5 Inadequately
5 How difficult was it for you?	1 Not difficult
	2 Quite difficult
	3 Neutral
	4 Difficult
	5 Too difficult
6 What were some of the challenges you faced?	
7 What was done well?	
8 Which areas of the training could be improved?	
9 Over all comments:	

## Abbreviations

ETT: Endotracheal tube
POCUS: Point of care ultrasound
NICU: Neonatal intensive care unit
AKUH: Aga Khan University Hospital
OSATS: Objective structured assessment of technical skills
SD: Standard deviation
SBME: Simulator based medical education
ERC: Ethical review committee
FAST: Focused assessment with Sonography for trauma
